# Chloroform in Obstruction of the Bowels from Spasms

**Published:** 1852-09

**Authors:** D. J. Cain


*Chloroform in Obstruction of the Bowels from Spasms.* By D. J. Cain, M. D.—Every physician meets, in the course of his practice, with cases of obstruction of the intestines, which has come on gradually or suddenly, generally, from some cause of irritation existing in them. The obstruction in these cases consists in spasmodic contraction of a portion, or of portions, of the intestines, generally the small. The plan of treatment which I formerly pursued was, to cease all attempts at forcing a passage by means of cathartics, if one or two brisk cathartics failed at the commencement, and to resort to opium freely, enemata of warm water, melted lard or butter, sweet oil, etc., the warm bath, fomentations to the abdomen, and other means of inducing relaxation. For more than two years past, I have used chloroform, as a more powerful agent than opium and its preparations, and as more certain in relaxing the muscular system. The chloroform, administered in inhalation, soon produces a greater or less degree of resolution, and, taking advantage of the relaxation thus effected, I give enemata, either stimulating, mucilaginous, or oily, which in a short time bring away fecal matter. The inhalation may be repeated as frequently as, in the judgment of the physician, the case demands.

Chloroform possesses the immense advantage over opium, of relieving effectually and promptly the pain, and in not leaving the bowels in a constricted state, the sedative effect soon passing off.

*Seven* cases have been thus treathd by me, with highly satisfactory results. In one case, only, have I experienced any difficulty in inducing the requisite degree of relaxation of the bowels. The subject of this case was very slightly susceptible of its influence; but the pain was completely relieved by frequent inhalations, and the obstruction gradually overcome.—*Charleston Med. Jour.*

